# Diagnostic accuracy of contrast-enhanced ultrasound versus duplex ultrasound and computed tomography angiography for endoleak detection after EVAR: a prospective comparative study

**DOI:** 10.1007/s40477-026-01182-4

**Published:** 2026-07-27

**Authors:** Wellington Gianoti Lustre, Libnah Leal, Vinicius Cardoso, Mateus Aves Borges Cristino, Carolina Dutra Queiroz Flumignan, Ronald Luiz Gomes Flumignan, Jorge Eduardo de Amorim, Luis Carlos Uta Nakano

**Affiliations:** 1 Division of Vascular and Endovascular Surgery, Universidade Federal de São Paulo, 754, Rua Borges Lagoa, Vila Clementino, São Paulo, SP 04038-001 Brazil; 2 Division of Vascular Surgery, Hospital Mater Dei, Belo Horizonte, Brazil; 3 Division of Vascular Surgery, Centro Universitario Sao Camilo, São Paulo, Brazil

**Keywords:** Abdominal aortic aneurysm, Endovascular aortic repair, Contrast-enhanced ultrasound, Duplex ultrasound, Computed tomography angiography, Diagnostic accuracy

## Abstract

**Background:**

Lifelong imaging surveillance after endovascular aneurysm repair (EVAR) is essential for endoleak detection but is limited by cumulative radiation and nephrotoxicity from computed tomography angiography (CTA). Contrast-enhanced ultrasound (CEUS) has emerged as a promising alternative, although prospective comparative data remain limited.

**Methods:**

In this prospective, single-centre diagnostic accuracy study, 30 consecutive patients (mean age 78.5 ± 7.1 years; 70% male) undergoing EVAR follow-up were evaluated with duplex ultrasound (DUS), CEUS (SonoVue®), and CTA within a 30-day interval. CTA served as the reference standard. Diagnostic performance metrics were calculated with 95% confidence intervals (CIs) according to STARD recommendations.

**Results:**

Endoleaks were identified in 11 patients (36.7%). CTA detected 8 cases (26.7%), CEUS 9 (30.0%), and DUS 4 (13.3%). CEUS demonstrated high sensitivity (87.5%; 95% CI 47.3–99.7%) and specificity (90.9%; 70.8–98.9%), with overall accuracy of 90.0% (73.5–97.9%). In contrast, DUS showed limited sensitivity (33.3%; 9.9–65.1%) despite perfect specificity (100%), yielding lower accuracy (73.3%). Inter-modality agreement was 83.3%.

**Conclusions:**

CEUS demonstrates high diagnostic accuracy for endoleak detection after EVAR and represents a robust, radiation-free alternative to CTA for longitudinal surveillance. Given its favourable safety profile and diagnostic performance, CEUS should be considered a first-line imaging modality in routine follow-up, with CTA reserved for equivocal findings or pre-interventional planning. These results support a paradigm shift toward CEUS-based surveillance strategies.

**Graphical Abstract:**

**Figure:**
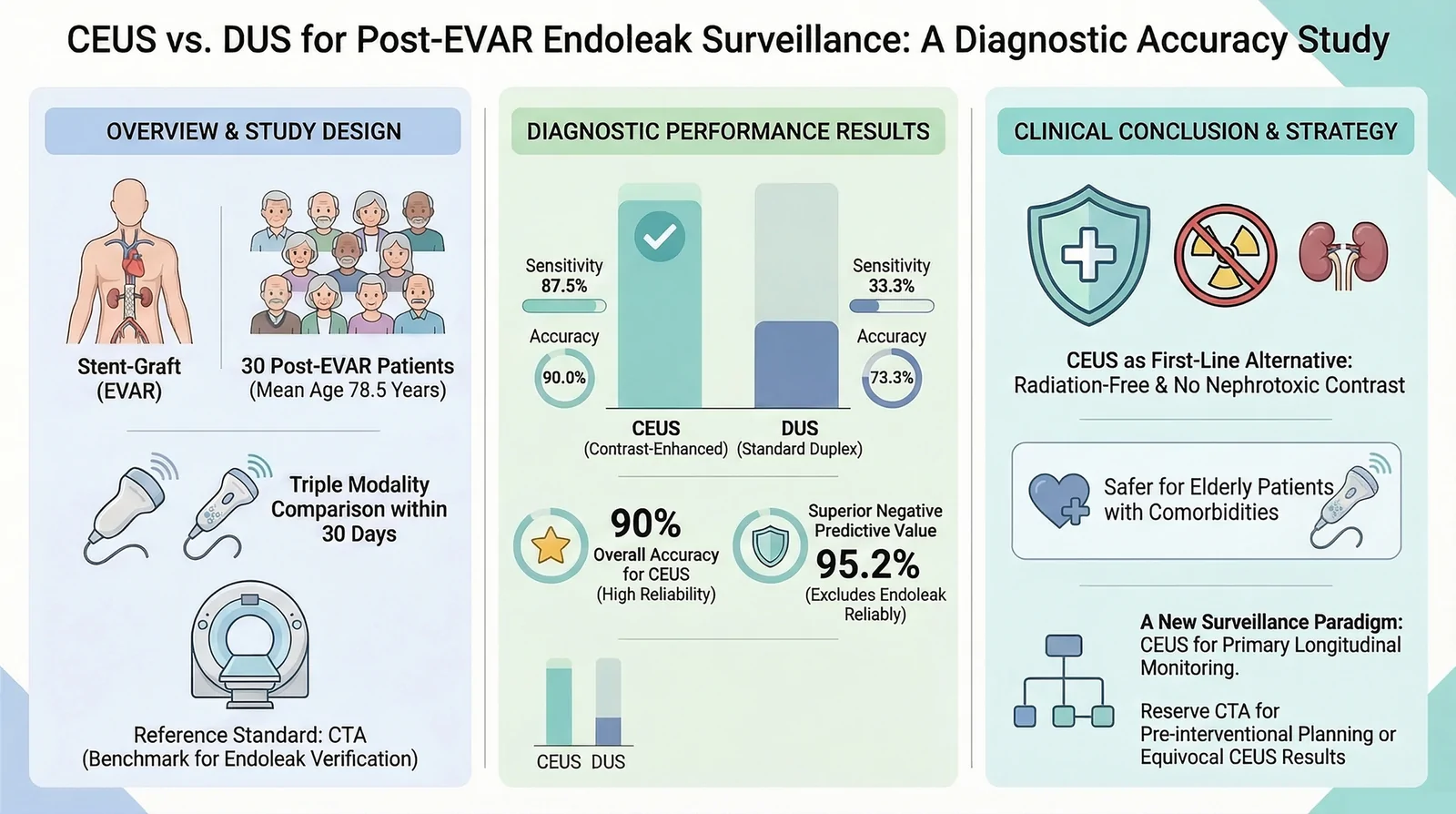

**Supplementary Information:**

The online version contains supplementary material available at https://doi.org/10.1007/s40477-026-01182-4.

## Introduction

Endovascular aneurysm repair (EVAR) has become the preferred treatment for infrarenal abdominal aortic aneurysms (AAA), owing to its lower perioperative morbidity and faster recovery compared with open surgical repair [1, 2]. Nevertheless, its long-term durability is limited by device-related complications, particularly endoleaks, which remain the leading cause of aneurysm sac enlargement and secondary intervention [3]. Consequently, lifelong imaging surveillance is mandatory to ensure the early detection and timely management of these complications [4].

Computed tomography angiography (CTA) is widely regarded as the reference standard for post-EVAR surveillance because of its high spatial resolution and excellent reproducibility [5]. However, repeated CTA examinations are associated with cumulative exposure to ionising radiation and iodinated contrast media, raising concerns regarding long-term safety, particularly in older patients with multiple comorbidities or impaired renal function [6]. These limitations have prompted the search for safer, more sustainable imaging strategies capable of maintaining high diagnostic accuracy while reducing patient burden [7].

Duplex ultrasound (DUS) is frequently used as a first-line imaging modality because it is widely available, non-invasive, and inexpensive. However, its diagnostic performance is highly operator-dependent and its sensitivity is limited, particularly for the detection of low-flow or occult endoleaks [8]. In contrast, contrast-enhanced ultrasound (CEUS), which employs intravascular microbubble contrast agents, enables real-time assessment of endograft perfusion and blood flow, improving the detection of low-velocity endoleaks without exposing patients to ionising radiation or nephrotoxic contrast agents [9–11].

Although growing evidence supports the use of CEUS for post-EVAR surveillance, prospective head-to-head comparisons with CTA remain limited, particularly in routine clinical practice [12]. Moreover, recent health economic analyses have suggested that CEUS-based surveillance strategies may be more cost-effective than CTA-centred follow-up pathways, further supporting their potential role in long-term monitoring.

The substantial epidemiological burden of AAA, together with the advanced age and high prevalence of comorbidities among affected patients, further underscores the need to optimise surveillance strategies that maximise diagnostic performance while minimising procedure-related risks [13].

The present study aimed to prospectively evaluate the diagnostic accuracy of CEUS and DUS compared with CTA for the detection of endoleaks following EVAR.

## Methods

### Study design and setting

This single-centre, cross-sectional diagnostic accuracy study was conducted in accordance with the *Standards for Reporting Diagnostic Accuracy Studies* (STARD) guidelines [14]. The study protocol was approved by the local Research Ethics Committee (Protocol No. 35898420.7.0000.5505). Data were prospectively collected from consecutive patients undergoing follow-up at the Division of Vascular and Endovascular Surgery of a quaternary university hospital between January and July 2024. Written informed consent was obtained from all participants before enrolment.

### Participants

Eligible participants were adults who met the following criteria: (1) diagnosis of an infrarenal abdominal aortic aneurysm (AAA); (2) previous EVAR performed between January and July 2024; (3) ongoing follow-up at the Division of Vascular and Endovascular Surgery; and (4) completion of duplex ultrasound (DUS), contrast-enhanced ultrasound (CEUS), and computed tomography angiography (CTA) within a maximum interval of 30 days.

Exclusion criteria included known hypersensitivity to sulfur hexafluoride contrast agent (SonoVue®), intracardiac shunts, severe pulmonary hypertension (systolic pulmonary artery pressure >90 mmHg), acute coronary syndrome, unstable angina, or inability to complete the scheduled imaging protocol. Patients who did not attend one or more scheduled imaging examinations after enrolment were excluded from the final analysis.

### Sample size calculation

The sample size was estimated using the method proposed by Flahault *et al.* for diagnostic accuracy studies [15]. Assuming an expected sensitivity of 90% for CEUS, based on a previous systematic review [16], a precision of ±15%, a 95% confidence level, and an anticipated endoleak prevalence of 35% in a long-term EVAR surveillance population, the minimum required sample size was 27 participants. To compensate for potential losses due to missed appointments or incomplete imaging, the recruitment target was increased to 30 patients.

### Index tests: DUS and CEUS

All ultrasound examinations were performed in the Vascular Ultrasound Unit by a single experienced vascular sonographer who had previously performed more than 100 EVAR surveillance examinations and was blinded to the CTA findings. Conventional ultrasound assessment was performed first and included B-mode imaging, colour Doppler, and spectral Doppler evaluation of the abdominal aorta, iliac arteries, and aneurysm sac. CEUS was subsequently performed during the same examination session.

CEUS was carried out using sulfur hexafluoride microbubble contrast (SonoVue®, Bracco Suisse SA, Geneva, Switzerland), administered as a 2–5 mL intravenous bolus through an 18- or 20-gauge peripheral venous catheter, followed by a saline flush. Images were acquired using contrast-specific pulse-inversion harmonic imaging at a low mechanical index (0.10–0.20) with real-time split-screen display. Flash-replenishment techniques were applied when necessary to optimise visualisation of contrast enhancement and endoleak perfusion. Dynamic cine loops and representative still images were stored for subsequent analysis. Endoleaks were classified according to the established reporting criteria described by White *et al.* [3].

### Reference standard: CTA

Computed tomography angiography (CTA) of the abdomen and pelvis was performed in the Department of Diagnostic Imaging using a standardised multiphasic protocol that included unenhanced, arterial, and delayed venous phases. All CTA examinations were independently interpreted by a board-certified radiologist who was blinded to the ultrasound findings. CTA served as the reference standard for all analyses of diagnostic performance.

### Statistical analysis

The diagnostic performance of CEUS and DUS was evaluated independently against the CTA reference standard. Sensitivity, specificity, positive predictive value (PPV), negative predictive value (NPV), and overall diagnostic accuracy were calculated with exact binomial 95% confidence intervals (CIs). Agreement with the reference standard was expressed as the proportion of cases in which the index test result (positive or negative for endoleak) was concordant with the CTA findings. Statistical analyses were performed using IBM SPSS Statistics for Windows, version 25.0 (IBM Corp., Armonk, NY, USA).

## Results

### Patient characteristics

**Fig. 1:**
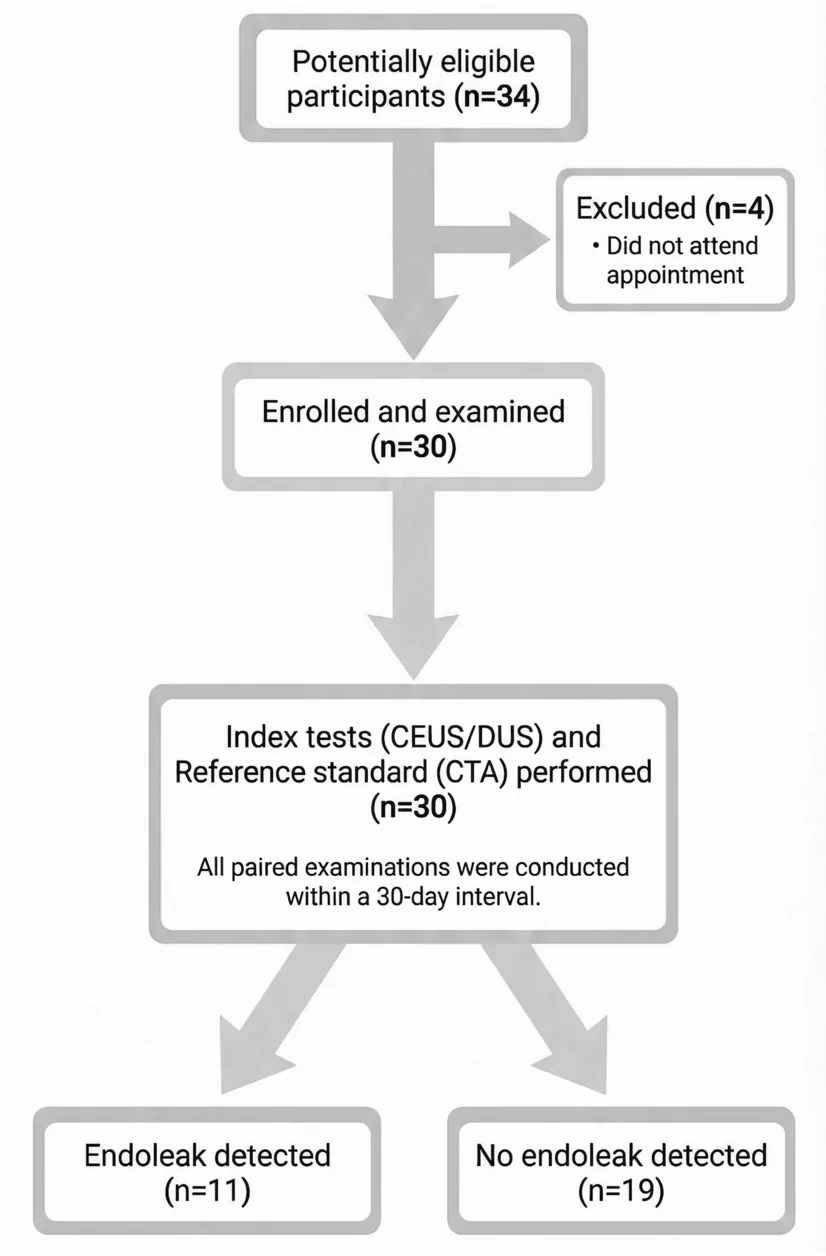
STARD flow diagram

Among the 34 eligible patients identified during the study period, 30 completed the full imaging protocol and were included in the final analysis (Fig. 1). Four patients were excluded because they did not attend their scheduled imaging appointments.

The mean age of the study population was 78.5 years (SD, 7.1; range, 66–95 years). Most participants were male (21/30, 70.0%) and self-identified as White (24/30, 80.0%). A history of smoking (current or former) was reported by 19 patients (63.3%). Hypertension was the most common comorbidity (25/30, 83.3%), followed by dyslipidaemia (14/30, 46.7%) and non-coronary cardiovascular disease (13/30, 43.3%). Overall, 13 patients (43.3%) had three or more comorbidities, and 3 (10.0%) had more than five. Detailed demographic and clinical characteristics are presented in Table 1.

**Table 1:** Demographic and Clinical Characteristics of the Study Population (*n* = 30)

Characteristic	n / Mean	% / SD	Range / *p*-value
Age (years)	78.48	± 7.12	66–95
Sex – Male	21	70.0%	–
Sex – Female	9	30.0%	–
Race – White	24	80.0%	–
Race – Black	4	13.3%	–
Race – Asian / Indigenous	2	6.7%	–
Ex-smoker	16	53.3%	–
Current smoker	3	10.0%	–
Non-smoker	11	36.7%	–
Hypertension	25	83.3%	–
Dyslipidaemia	14	46.7%	–
Non-coronary cardiovascular disease	13	43.3%	–
Diabetes mellitus	8	26.7%	–
Coronary artery disease	5	16.7%	–
Chronic kidney disease	2	6.7%	–
Patients with ≥ 3 comorbidities	13	43.4%	–
Mean comorbidities / patient	2.80	± 0.92	–
Time EVAR → imaging: CTA (years)	5.62	± 4.62	–
Time EVAR → imaging: DUS/CEUS (years)	5.43	± 4.74	–

*DUS* duplex ultrasound, *CEUS* contrast-enhanced ultrasound, *CTA* computed tomography angiography, *SD* standard deviation.

The mean interval between EVAR and imaging was 5.62 years (SD, 4.62) for CTA and 5.43 years (SD, 4.74) for DUS/CEUS, indicating comparable follow-up timing between imaging modalities.

### Endoleak detection and inter-modality agreement

Nineteen of the 30 patients (63.3%) showed no evidence of endoleak on any imaging modality. Overall, endoleaks were detected in 11 patients (36.7%). CTA detected 8 endoleaks (26.7%), CEUS detected 9 (30.0%), and DUS detected 4 (13.3%).

**Fig. 2:**
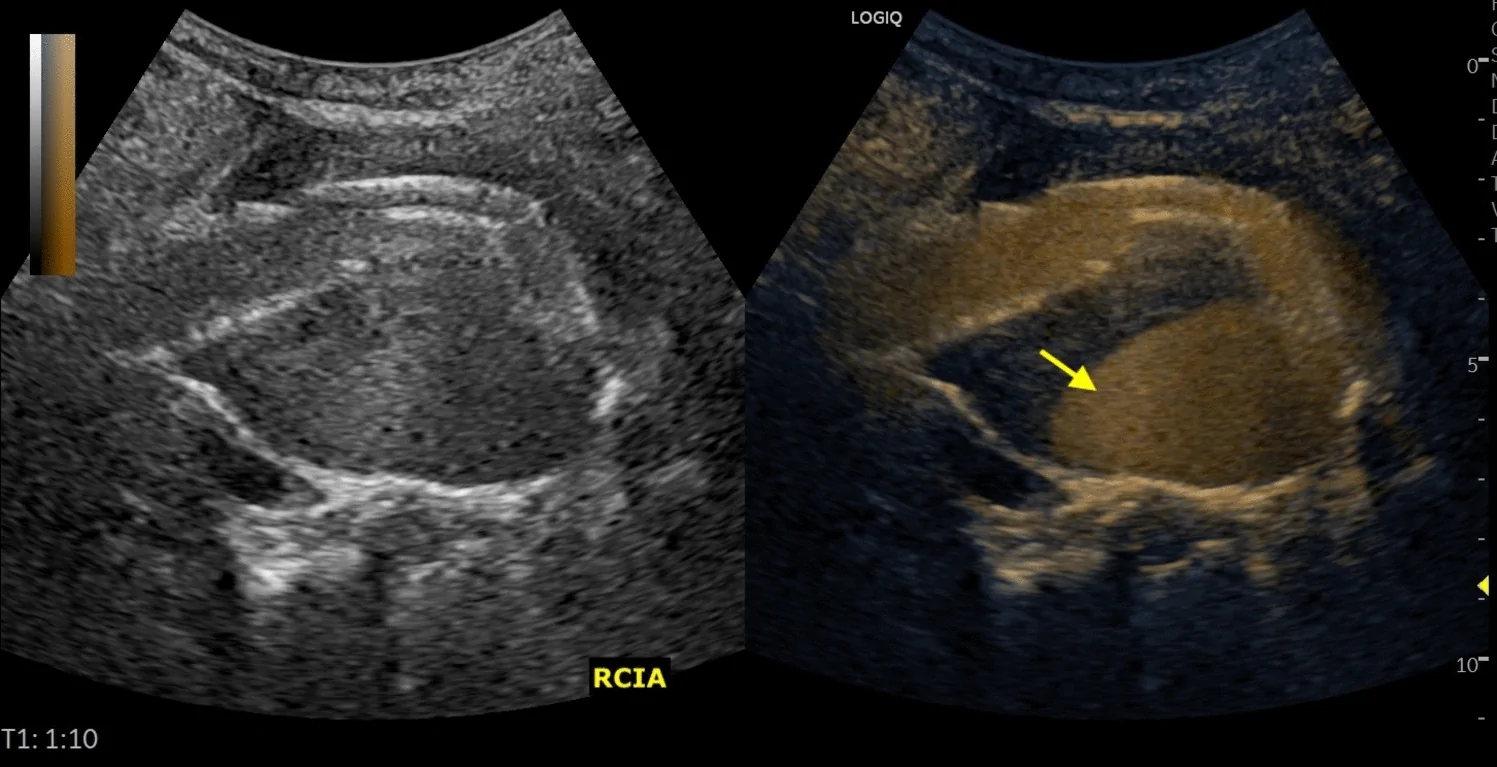
Contrast-enhanced ultrasound (CEUS) of endoleak detection after endovascular aneurysm repair (EVAR). Split-screen showing B-mode ultrasound (left) and contrast-specific low–mechanical index imaging (right) after intravenous sulfur hexafluoride microbubbles (SonoVue^®^). Enhancement within the aneurysm sac (arrow) indicates a type III endoleak not detected on colour Doppler ultrasound (DUS)

A representative type III endoleak identified by contrast-enhanced ultrasound (CEUS) is shown in Fig. 2, demonstrating focal intraluminal microbubble enhancement within the aneurysm sac, consistent with endograft disruption. The dynamic contrast-filling pattern and flow characteristics are illustrated in the Supplementary Video (Appendix 1).

Among the 11 patients with an endoleak, all three imaging modalities were concordantly positive in 4 cases (36.4%), whereas CTA and CEUS were concordantly positive in 7 cases (63.6%). Overall agreement between the imaging modalities was observed in 25 of 30 patients (83.3%), with discordant findings in the remaining 5 patients (16.7%). Detailed agreement data are summarised in Table 2.

**Table 2:** Endoleak Detection Rates and Method Concordance (n = 30)

Finding	CTA	CEUS	DUS
Negative for endoleak (all 3 methods)	19 (63.4%)	19 (63.4%)	19 (63.4%)
Endoleak detected	8 (26.7%)	9 (30.0%)	4 (13.3%)
3-method concordance (endoleak positive)	36.4%	–	–
CTA vs CEUS concordance	63.6%	–	–
Overall inter-method concordance	83.3%	–	–

*CTA* computed tomography angiography, *DUS* duplex ultrasound, *CEUS* contrast-enhanced ultrasound.

### Diagnostic performance

CEUS demonstrated a sensitivity of 87.5% (95% CI, 47.3–99.7%), specificity of 90.9% (95% CI, 70.8–98.9%), positive predictive value (PPV) of 77.8% (95% CI, 47.6–93.1%), negative predictive value (NPV) of 95.2% (95% CI, 76.1–99.2%), and overall diagnostic accuracy of 90.0% (95% CI, 73.5–97.9%). These findings indicate that a negative CEUS examination was associated with a high probability of excluding endoleak, correctly ruling out disease in approximately 19 of 20 cases.

DUS demonstrated perfect specificity (100.0%; 95% CI, 81.5–100.0%) and PPV (100.0%; 95% CI, 39.8–100.0%), indicating that positive DUS findings were consistently associated with the presence of endoleak. However, sensitivity was limited (33.3%; 95% CI, 9.9–65.1%), and the NPV was 69.2% (95% CI, 60.1–77.1%), suggesting that a negative DUS examination alone is insufficient to exclude endoleak. Overall diagnostic accuracy was 73.3% (95% CI, 54.1–87.7%). Detailed diagnostic performance measures are presented in Table 3.

**Table 3:** Diagnostic Performance of CEUS and DUS for Endoleak Detection versus CTA Reference Standard

Metric	CEUS	95% CI	DUS	95% CI
Sensitivity	87.5%	47.3–99.7%	33.3%	9.9–65.1%
Specificity	90.9%	70.8–98.9%	100.0%	81.5–100.0%
PPV	77.8%	47.6–93.1%	100.0%	39.8–100.0%
NPV	95.2%	76.1–99.2%	69.2%	60.1–77.1%
Overall Accuracy	90.0%	73.5–97.9%	73.3%	54.1–87.7%

*PPV* positive predictive value, *NPV* negative predictive value, 95% CI = 95% confidence interval, *CEUS* contrast-enhanced ultrasound, *DUS* duplex ultrasound.

## Discussion

**Fig. 3:**
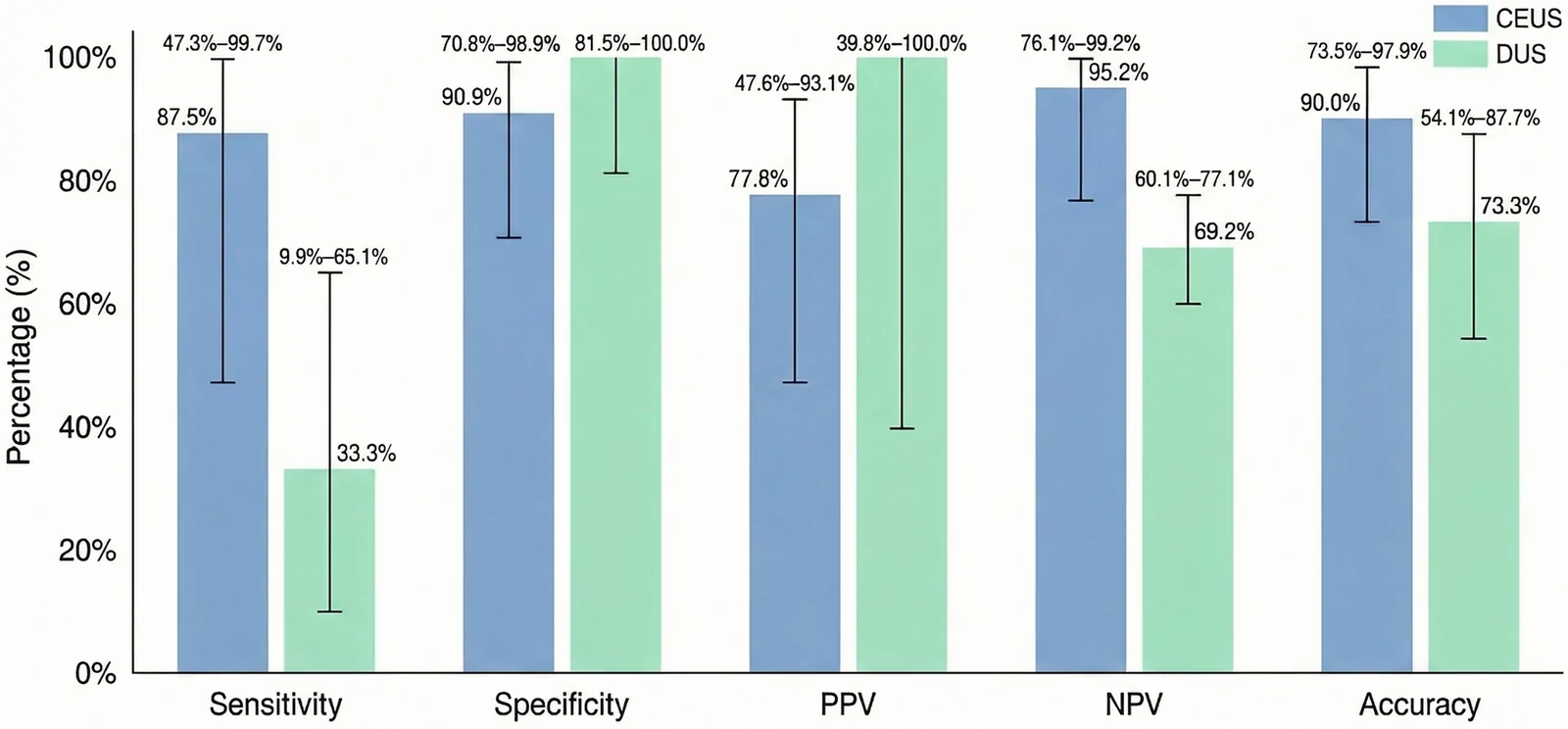
Comparative diagnostic performance of contrast-enhanced ultrasound (CEUS) and duplex ultrasound (DUS) using computed tomography angiography (CTA) as the reference standard (95% confidence intervals)

This prospective diagnostic accuracy study conducted at a high-volume Brazilian quaternary referral centre found that CEUS achieved high diagnostic accuracy for the detection of endoleaks following EVAR, with an overall accuracy of 90.0%, sensitivity of 87.5%, and NPV of 95.2%. In contrast, DUS demonstrated markedly lower sensitivity (33.3%) despite excellent specificity, supporting the use of CEUS as the preferred ultrasound-based imaging modality for routine post-EVAR surveillance Fig. 3.

The diagnostic performance of CEUS observed in the present study is consistent with the highest level of available evidence. A Cochrane systematic review by Abraha *et al.* reported pooled sensitivity and specificity of 94% and 95%, respectively, supporting the use of CEUS as a primary surveillance modality with CTA reserved for positive or inconclusive findings [11]. Similarly, Karaolanis *et al.* reported pooled endoleak detection rates of 96.7% for CEUS and 92.8% for CTA, suggesting that CEUS may outperform CTA in selected clinical scenarios [9]. Harky *et al.* also demonstrated significantly higher sensitivity for CEUS without loss of specificity [10], while the prospective study by Brkláček *et al.* reported sensitivity of 91.2% and specificity of 100%, closely matching our findings [11].

The principal advantage of CEUS lies in its ability to depict real-time blood flow, facilitating the detection of type II and other low-flow endoleaks that may be missed during the limited acquisition window of arterial-phase CTA [2, 9, 11]. This physiological advantage is reflected in current recommendations. The 2024 ESVS Guidelines recognise CEUS as a diagnostic modality with performance comparable to CTA for post-EVAR surveillance [3], and the ESSA trial likewise demonstrated high diagnostic agreement with CTA while providing additional haemodynamic information [8].

Beyond diagnostic accuracy, CEUS offers important practical advantages. By avoiding ionising radiation and iodinated contrast media, it represents an attractive option for long-term surveillance, particularly in older patients and those with chronic kidney disease or multiple comorbidities. Sulfur hexafluoride microbubble contrast (SonoVue®) is eliminated through pulmonary exhalation and is not associated with nephrotoxicity [17]. In addition, recent health economic analyses suggest that CEUS-based surveillance strategies may be more cost-effective than CTA-centred follow-up because of lower imaging costs and fewer contrast-related adverse events.

Our findings also confirm the limited role of conventional DUS as a standalone surveillance modality. Although specificity was 100%, sensitivity was only 33.3%, indicating that a substantial proportion of endoleaks would not be detected by DUS alone. These findings are consistent with previous evidence. Mirza *et al.* reported a pooled sensitivity of 0.77 with considerable between-study heterogeneity [8], whereas Karadag *et al.* reported a sensitivity of 63.6% in a prospective cohort [19]. Collectively, these data suggest that DUS is better suited as an initial triage or adjunctive examination than as the primary imaging modality for long-term surveillance [20, 21].

An additional strength of the present study is that it provides prospective evidence from a high-volume Latin American centre, a region that remains underrepresented in the current literature. The clinical characteristics of our cohort, including the high prevalence of hypertension, smoking, and dyslipidaemia, are consistent with the established epidemiology of AAA [13]. These findings may therefore be particularly relevant for healthcare settings where repeated CTA is constrained by limited resources or concerns regarding cumulative contrast exposure [18].

Several limitations should be acknowledged. Although the sample size was determined a priori and was adequate for the primary analysis, the relatively small number of patients resulted in wide confidence intervals, particularly for sensitivity estimates. Larger multicentre studies are needed to confirm these findings and to evaluate diagnostic performance according to endoleak subtype. Although data collection was prospective, patient identification relied on retrospective screening of institutional records, introducing the possibility of selection bias. The cross-sectional design precluded assessment of the longitudinal evolution of endoleaks, including delayed occurrence and spontaneous resolution. Furthermore, all ultrasound examinations were performed by a single experienced operator. Although this approach minimised measurement variability, it limits assessment of interobserver reproducibility. Finally, the predominance of White patients reflects the local referral population and may limit the generalisability of the findings to more ethnically diverse populations.

## Conclusions

CEUS using sulfur hexafluoride microbubbles (SonoVue®) demonstrates high diagnostic accuracy for the detection of endoleaks after EVAR and represents a reliable, radiation-free alternative to CTA for longitudinal surveillance. Its high negative predictive value supports its potential role as a first-line imaging modality for excluding clinically relevant endoleaks and reducing the need for repeated CTA examinations. Conventional DUS alone showed limited sensitivity and appears insufficient as a standalone surveillance strategy. These findings support the integration of CEUS into post-EVAR follow-up pathways, with CTA remaining appropriate for equivocal findings, suspected complications requiring further characterisation, or pre-interventional planning.

## Supplementary Information

Below is the link to the electronic supplementary material. The data supporting the findings of this study are available from the corresponding author upon reasonable request.

Supplementary file1 (MP4 2260 KB)

## Data Availability

The data supporting the findings of this study are available from the corresponding author upon reasonable request.
